# Fungal Pathogens of Maize Gaining Free Passage Along the Silk Road

**DOI:** 10.3390/pathogens7040081

**Published:** 2018-10-11

**Authors:** Michelle E. H. Thompson, Manish N. Raizada

**Affiliations:** Department of Plant Agriculture, University of Guelph, Guelph, ON N1G 2W1, Canada; mthomp15@uoguelph.ca

**Keywords:** maize, silk, style, ear rot, fungal pathogen, mycotoxin, *Fusarium graminearum*, *Fusarium verticillioides*, *Aspergillus flavus*, *Ustilago maydis*

## Abstract

Silks are the long threads at the tips of maize ears onto which pollen land and sperm nuclei travel long distances to fertilize egg cells, giving rise to embryos and seeds; however fungal pathogens also use this route to invade developing grain, causing damaging ear rots with dangerous mycotoxins. This review highlights the importance of silks as the direct highways by which globally important fungal pathogens enter maize kernels. First, the most important silk-entering fungal pathogens in maize are reviewed, including *Fusarium graminearum*, *Fusarium verticillioides*, and *Aspergillus flavus*, and their mycotoxins. Next, we compare the different modes used by each fungal pathogen to invade the silks, including susceptible time intervals and the effects of pollination. Innate silk defences and current strategies to protect silks from ear rot pathogens are reviewed, and future protective strategies and silk-based research are proposed. There is a particular gap in knowledge of how to improve silk health and defences around the time of pollination, and a need for protective silk sprays or other technologies. It is hoped that this review will stimulate innovations in breeding, inputs, and techniques to help growers protect silks, which are expected to become more vulnerable to pathogens due to climate change.

## 1. Introduction

Maize (*Zea mays* ssp. *mays*) is a staple crop used around the globe for food, animal feed, biofuel, and bioproducts [1]. Maize is grown in both temperate and tropical regions including Sub-Saharan Africa and Latin America, and is one of the world’s top three most important food crops [2]. In the United States alone it is worth US $75 billion annually to the economy [3,4]. In 2018, maize made up 96% of U.S. feed grain production [5]. Maize supports a diversity of farmers, not only large industrial farmers who grow it for feed and bioproducts, but also smallholders who rely on it as a direct form of nourishment. Due to the global importance of the crop, maize diseases cause hefty economic, nutritional, and livelihood impacts. Ear rots are especially problematic, as they directly affect the grain. Of particular importance are ear rot fungi that produce mycotoxins, which can be present at dangerous levels in the grain with and without visible symptoms. Mycotoxins accumulate in kernels preharvest but can further increase during storage. This is particularly problematic for many farmers in the tropics, where financial and environmental challenges exacerbate mycotoxin accumulation. Fiscal challenge leads to inadequate storage infrastructure and a lack of affordable, validated mycotoxin testing, and the high humidity and temperatures in the tropics further create an advantageous habitat for toxigenic fungi [6,7]. In turn, this leads to mycotoxins in food and feed.

**Figure 1:**
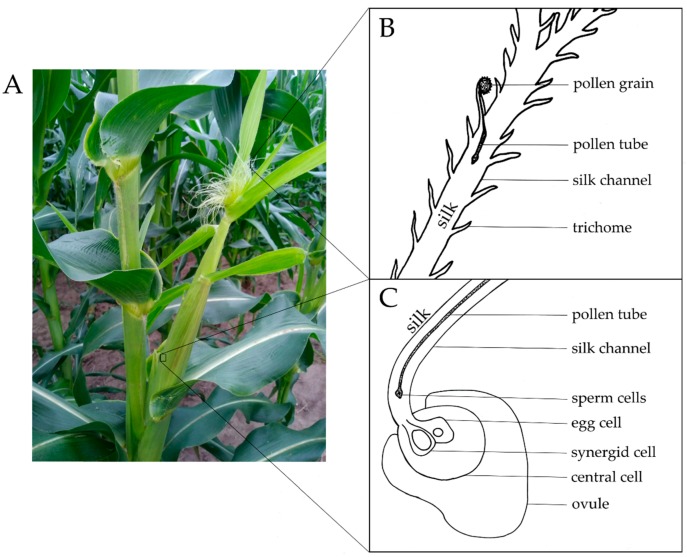
Maize silks and their normal function. (**A**) Maize silks emerging on a young cob. (**B**) Initial pollen entry onto a hair (trichome) of an individual silk: A pollen grain lands on the exposed silk tissue, from which a pollen tube penetrates the silk channel. Silk hairs, or trichomes, help to guide the pollen into the silks. (**C**) The exit of the sperm nuclei from the pollen tube-silk complex into the ovule leading to double fertilization: The pollen tube reaches the base of the silk, transporting the two sperm cells to their destination in the ovule. The pollen tube is received by the synergid cell within the ovule. One sperm cell will fertilize the egg cell, while the other will fertilize the central cell.

Ear rot pathogens can enter maize kernels through various routes including wounds caused by insects or systemically from the stalk [8,9,10]. Perhaps underappreciated is that some of the most devastating ear rot fungi have a common feature—they enter via the style, which in maize is a thread-like channel called the silk (Figure 1). In maize, pollen from the tassel at the top of the plant is dispersed by wind to exposed, receptive silks on an ear lower on the stalk of the same plant (self-pollination) or neighbours (cross-pollination) [11]. Pollen lands on stigmatic hairs or other epidermal cells on exposed silks where it subsequently germinates to produce an elongated pollen tube, which grows through the silk to facilitate double fertilization [11,12] (Figure 1). Each silk leads to an individual ovule, hence the silk is the female reproductive tract of maize, comparable to the uterus and the Fallopian tubes in humans [12]. Hundreds of kernels per ear require hundreds of silk channels. Furthermore, ancient farmers in Mexico selected for large cobs surrounded by protective husk leaves, under which silks must grow and emerge [13]. Thus, modern maize silks are hyperelongated and, interestingly, amongst the fastest growing tissues in nature, expanding at a rate of 1 to 3 mm per hour [14,15]. Moreover, as silks are a water-dense, soft, and nutrient-rich tissue protected within tightly wrapped husk leaves [16], they provide a good substrate for fungi to temporarily colonize prior to the more permanent home in the seed. What is critical to this review is that for maize to receive the pollen, the silks must be exposed to the external environment, and hence also serve as doorways to opportunistic environmental pathogens [11]. Silk hairs, or trichomes, guide pollen tubes into the body of the silk, and similarly lead fungi through the same route [17]. It is interesting to note that in humans, the female reproductive tract, similarly exposed to the environment, is also prone to fungal pathogens (e.g., *Candida albicans*) [18].

The purpose of this review is to highlight the importance of the silk channel as the direct highway by which globally important fungal pathogens enter maize kernels. The paper has four objectives: (1) to review the most important silk-entering fungal pathogens in maize; (2) to compare the modes that fungal pathogens use to invade the silk route; (3) to review the innate silk defence system and current strategies for fighting silk-entering fungi in order to (4) direct future research and lead to targeted strategies to help growers protect silks.

## 2. Globally Important Pathogens That Infect Maize Silks and Subsequently Grain

This section will review the most globally important fungal pathogens of maize that enter grain through the silks, with a special focus on the literature related to the susceptibility of silk tissue. These pathogens are summarised in Table 1.

### 2.1. Gibberella Ear Rot

*Fusarium graminearum* is the fungus which causes Gibberella ear rot (GER) [19] (Table 1). *F. graminearum* produces a suite of mycotoxins, most notably deoxynivalenol (DON, or vomitoxin) and zearalenone, in grain before and after harvest [20,21,22]. DON blocks the synthesis of proteins and DNA in mammals, can result in symptoms such as vomiting, diarrhea, headaches, and fever, while zearalenone is an estrogen analogue which disturbs hormonal regulation [23,24,25,26,27,28]. DON is not degraded by normal cooking temperatures [28], and in 2009, 80% of 1-year-old children in the Netherlands consumed more than the maximum daily intake of DON [29]. It is unknown how chronic, low-level intake of mycotoxins affects humans [30].

Since *F. graminearum* produces dangerous mycotoxins, it is important to understand the path it takes to invade the grain. The airborne spores of *F. graminearum*, originating from decomposing crop residues, transmit the fungus to subsequent growing seasons [31]. It takes 7 to 15 days for conidia to germinate on silks and for hyphae to grow through the silks to reach the young grain [17]. Similar to the entry of pollen, *F. graminearum* does not necessarily need to land on the distal silk tip, but can infect along the entire exposed length of a silk [11,32]. Hyphae travel through the silk parenchyma, vascular tissue, or pollen tube transmitting tissue without preference [17]. The fungus can also exit the individual silk it has colonised to access other parts of the ear [17]. It has been reported that ears are more susceptible to *F. graminearum* after silk emergence, logically because they become exposed to the environment, but once silks have dried, *F. graminearum* cannot travel through them [33,34]. However, Incremona et al. have observed hyphae growing in silks when inoculated at senescence [32]. They suggest that *F. graminearum* is hemi-biotrophic.

When breeding for resistance to GER, the suggested period for inoculation is 4 to 7 days after silk emergence [33]. Silks are most susceptible to *F. graminearum* infection after pollination, at the very beginning of senescence when they begin to turn brown [35]. Pollen can increase germination and growth of *F. graminearum* and *F. verticillioides* in exposed silks (meaning outside of the husk) [17,36].

Incremona et al. showed that silks inoculated with *F. graminearum* during pollination had lower infection rates than either those inoculated prior to pollination or during senescence [32]. Inoculation was done under conditions that were suboptimal for infection. Could the lower infection rate be due to a heightened plant defence system at the time of pollination? This study did not proceed through to grain filling, rather it only measured silks for infection; it would be useful to know if the grain itself was protected.

The cob has higher mycotoxin accumulation than the kernels [37,38], which indicates that fungus may subsequently use the cob as a route of passage to access more kernels [17,39]. High toxin levels in the cob could pose a greater risk to livestock which consume feeds containing whole cobs, such as silage. Therefore, if a *F. graminearum* spore is allowed passage along an individual silk, it can lead to infection throughout the entire cob.

### 2.2. Fusarium Ear Rot

*F. verticillioides*, *F. proliferatum*, and *F. subglutinans* are the causal agents of Fusarium ear rot [40] (Table 1). *F. verticillioides* is of particular concern, as it produces the carcinogenic mycotoxin fumonisin B_1_ [41]. Fumonisins cause liver and esophageal cancers in humans [41,42]. Maize tissues around the world by far contain the highest amounts of fumonisins compared to other crops [41]. Even when farmers stored maize-based feed on a raised place, fumonisin B_1_ was observed to contaminate 86% of samples tested in Kenya in 2006 [43].

While *F. verticillioides* produces harmful fumonisins, it also serves as an endophytic fungus in maize [40,44]. It can help manage the fungal pathogen *Ustilago maydis*, and it is an endophytic competitor to *F. graminearum* [41,45,46]. *F. verticillioides* is antagonistic to *U. maydis*, decreasing the biomass of *U. maydis* and increasing its metabolite concentration per unit of biomass [47]. However, the literature is lacking information about the interactions between these fungal species in silk tissue. The economic impact of *F. verticillioides* is multifaceted, as the fungus maintains complex relationships with other pathogens. It is considered less aggressive than *F. graminearum*, but fumonisins are still a concern [48].

*F. verticillioides* spores can land on the silks directly, or land on the male inflorescence, the tassel, and use pollen as a vector to reach the silks where they germinate [49]. Wind speed, precipitation, and humidity during silking impact subsequent fumonisin concentrations [49,50]. Although pollen-assisted transmission is possible, silks are most susceptible at 4 to 6 days after pollination [51]. While Duncan and Howard did not see evidence of *F. verticillioides* reaching grain via the silk route, this may be due to the inoculation method; silks were sprayed once at eight days after pollination, which is beyond the most susceptible period [36]. Munkvold et al. have shown silk inoculation to be the most effective route to produce *F. verticillioides*-infected grain, which is why it is commonly used as a selective treatment in breeding programs [9]. *F. verticillioides* hyphae have been observed growing along the epidermis of silks, but not necessarily through the interior of silks [52].

Like *F. graminearum*, pollen can increase germination and growth of *F. verticillioides* in the portion of silks that is outside the husk [36]. This observation calls for further research into the molecular factors that make certain fungi so pollen-philic. Is it that pollen supplies extra nutrients to the fungi, or that the innate silk defences are reduced near pollen grains?

### 2.3. Aspergillus Ear Rot

*Aspergillus flavus* and *Aspergillus parasiticus* are the fungi which cause Aspergillus ear rot (Table 1). *Aspergillus* fungi comprise perhaps the most harmful fungal contaminations of food, depositing teratogenic and mutagenic mycotoxins [21,42]. *A. flavus* is the more prevalent causal agent of Aspergillus ear rot in corn; for vulnerable people in the tropics, it is a leading source of dietary carcinogens including aflatoxins [23]. When examining climate change trends, aflatoxin accumulation in maize is expected to cost the U.S. up to US $1.68 billion per year [3]. While acute aflatoxicosis poisons humans, the greater concern is chronic aflatoxicosis which causes liver cancer [53]. Aflatoxins consumed by livestock can continue through the food chain to humans, making chronic aflatoxicosis an even more imminent threat [43].

It can take 4 to 13 days for *A. flavus* to move down the silks to their base nearest the ovules [54], which is the reason that in studies such as by Peethambaran et al., fungal inoculation was performed at seven days after mid-silking to assess the infection response [55]. However, the number of days after silk emergence may not be the best measure of a susceptibility period, since susceptibility is impacted by weather and cultivar [54]. Marsh and Payne used silk colour (green-yellow, yellow-brown, and brown) to address the silk “stages”, and found that inoculating yellow-brown silks resulted in high fungal growth [54]. Spores can survive on young silks, but *A. flavus* grows more vigorously after pollination when silks begin to senesce [54]. The high susceptibility of silks that are yellow-brown in colour may be due to the fact that the silks have diminished defences at senescence, but still retain sufficient nutrients for fungus to feed upon. Indeed, *A. flavus* appears to be saprotrophic and weakly parasitic, although early senescent, yellow-brown silks are thought to be most-susceptible, with high humidity and moisture; *A. flavus* can even colonize four-week-old brown silks [8].

Longer silk channels (defined as the distance from the cob tip to the tip of the husk where silks are first exposed) at pollination have been associated with lower aflatoxin levels [56]. This may be the result of a smaller portion of each silk being exposed beyond the husk tip, meaning less surface area for *A. flavus* to land on, and a longer tract of silk for the hyphae to traverse [56].

Lastly, *A. flavus* does not often infect the cob pith [54], however, like *F. graminearum*, *A. flavus* can travel through the cob to infect other kernels [57], and hence silk infection can lead to greater consequences.

### 2.4. Corn Smut

*Ustilago maydis*, also known as *Ustilago zeae* and *Ustilago zeae mays*, is a biotrophic fungus that causes Corn Smut (Common Smut, Boil Smut) [58] (Table 1). It can enter the plant at various sites, including silks which results in large fungal growths (galls) on the ear [58,59,60]. However, infection by *U. maydis* is not exclusively viewed in a negative light by local peoples; *Huitlacoche* or “maize mushrooms” are ear galls caused by Corn Smut which are eaten as a delicacy in Mexico, Central America, and the United States [59,60]. Inadvertent infection harms the grain, but *Huitlacoche* farmers intentionally infect maize for profit, garnering up to $40 kg^−1^ for the galls [61,62]. Although the *U. maydis* component of *Huitlacoche* is not thought to contain mycotoxins, the galls can facilitate infection with dangerous mycotoxin-producing pathogens including *A. flavus* and *F. graminearum* [20,63]. Interestingly, *Huitlacoche* contains antimutagenic substances [64], which begs the question whether these help counter mutagens introduced by other pathogens.

Silks are susceptible to *U. maydis* infection from tissue emergence to 8 to 14 days thereafter, with incidence decreasing dramatically over time [60]. The most-severe infections resulted from inoculation at 4 to 8 days after mid-silking (defined as silks emerged on 50% of plants), with the maximum occurrence at six days after mid-silking [61]. The suggested inoculation period for selective breeding for/against infection is 2 to 4 days after silk emergence [60].

In contrast to the aforementioned affinity of *Fusarium* for pollen, pollen is a beneficial factor in the fight against Corn Smut. Pollinated ears experience far less fungal growth in comparison to unpollinated ears [61,65]. The presence of pollen shortens the susceptibility period by 1 to 4 days and decreases the incidence of galls by up to 50% [60]. Similar effects have been noted in pearl millet [60]. Furthermore, stress (for example due to inclement weather), which can cause anthesis and silk emergence to occur asynchronously and thus reduce successful pollination, has been shown to result in high levels of infection in maize hybrids that are normally resistant [60]. This observation supports the theory that pollination prevents infection. An interesting hypothesis is that the pollen-associated decline in infection may be due to pollination accelerating the senescence of silks which would otherwise facilitate *U. maydis* transmission. Unlike *A. flavus*, *U. maydis* cannot colonize dead tissue, so the abscission zone at the base of pollinated silks may prevent *U. maydis* from reaching the kernels [66]. Maize genetics dictate the speed of abscission, and hence host genotypes with faster abscission speeds are expected to have better resistance to Corn Smut [67].

At the opposite end of the silk developmental spectrum, there is another relationship between silk age and the incidence of *U. maydis* infection, caused by hybrids sometimes having different silk emergence patterns [60]. The time from onset of silk emergence to completion differs amongst maize genotypes; longer emergence periods mean that there are at least some unpollinated silks present for a longer time [60] which presumably increases susceptibility to infection by *U. maydis*.

### 2.5. Diplodia/Stenocarpella Ear Rot

The last silk-entering fungal ear rot pathogen that will be discussed is Stenocarpella ear rot (Diplodia ear rot). This disease is caused by *Stenocarpella maydis*, previously known as *Diplodia maydis* or *Diplodia zeae* [68] (Table 1). This mould produces diplodiatoxin, which can cause diplodiosis, a deadly neural disorder in livestock [69]. This pathogen is reported to have many infection routes, including through the silks [69,70]. In resistant cultivars, silk is the most susceptible tissue [71]. The silk entry route is poorly studied for *S. maydis*, since infection through the husk and ear shank is much more common and economically relevant [70,72]. Further research into the silk-related mechanisms may provide insight into this disease.

## 3. Commonalities and Differences Amongst the Silk-Entering Pathogens

This section aims to compare the modes of the five fungal pathogens that use the silk route and identify gaps in research. Commonalities may direct future research and strategies to help growers to protect silks. Given their shared taxonomy, *F. graminearum* and *F. verticillioides* have many commonalities. For example, the same silk inoculation techniques can be used for both pathogens, demonstrating similar infection strategies [73]. A commonalty between four of the pathogens, *F. graminearum*, *F. verticillioides*, *A. flavus*, and *U. maydis*, is that all grow at least partially on living plants and thrive on plant tissue that is beginning to decay. Evidence for this statement is based on the following observations: First, *F. graminearum*, *F. verticillioides*, and *U. maydis* have been identified as partially biotrophic parasites [32,51,60]. Second, *A. flavus* has been referred to as weakly parasitic, which likewise involves feeding on living tissues [8]. Third, *F. graminearum*, *F. verticillioides*, *A. flavus*, and *U. maydis* all have saprotrophic abilities [8,35,36,60]. And finally, *A. flavus* and *F. graminearum* infect senescent silks [8,32]. The affinity of these pathogens for decaying plant tissue correlates to a silk’s most susceptible period, which is generally after pollination (in the case of *F. graminearum*, *F. verticillioides*, and *A. flavus*), as silks begin to senesce [35,51,54]. Early silk senescence provides a perfect environment for the fungi to access a silk’s nutrients and take advantage of a drop in natural silk defences that precedes tissue death, to permit hijacking of this route in order to infect the grain.

With respect to commonalities related to the impact of pollination on silk infection, outside of the husk leaves (i.e., exposed silk tips or harvested silks in vitro) pollination has been shown to increase the growth of the *Fusarium* pathogens, *F. graminearum*, and *F. verticillioides* [17,36]. There are gaps in research concerning *A. flavus* pollination interactions, but it is interesting to speculate that pollination may likewise improve *A. flavus* growth, given the high susceptibility of postpollination silks [54]. Pollination, a promoter of ear rots, cannot be avoided, but other protective strategies to combat all of the above Fusarium species need to be employed at the time interval surrounding pollination. Whilst pollination increases the growth of these fungal pathogens in exposed silks, it would be useful to know more about the growth of fungi within the covered portion of silks. The disease symptoms of GER progress from the exposed tip of the cob to the protected base [37]. This downward progression is in part due to the proximity of the tip to the environment [37]. Additionally, the lengths of silk that pathogens must traverse are shorter for kernels near the tip of the cob [37]. Given this observation, the greatest impact against GER would occur by directing disease-suppression strategies to the ear tip. As an important aside, it is interesting to note that pollination is also correlated with changes to the innate plant defence system. The underlying reason may be that flavonoid antioxidant capacity decreases as silks mature, which may contribute to the increase in ear rot susceptibility after pollination [16]. Levels of the lepidopteran insecticide, maysin, a flavonoid C-glycoside, also deplete after pollination [74,75], which is at the same stage that silks are most susceptible to the fungal pathogens described above. This observation makes one question whether maysin also has antifungal properties which contribute to this timing. Widstrom et al. observed silk maysin content and aflatoxin contamination but did not detect a correlation [76]. It would be interesting to examine silk maysin and the different silk-entering fungi in vitro to determine if maysin directly or indirectly influences fungal growth and aflatoxins.

The susceptible period and effect of pollination are where the literature begins to differentiate *U. maydis* from the other ear rot pathogens. The most susceptible period for *U. maydis* infection is close to silk emergence, with the maximum occurring six days after mid-silking [61]. Furthermore, pollination decreases infection, likely due to the formation of the abscission zone [61,65,66]. Thus, when *U. maydis* is a threat, timely pollination is especially important. Given that pollination is not only unavoidable for a commercial farmer but rather necessary for grain production, desiring efficient pollination to decrease Corn Smut is a realistic aspiration. *U. maydis* also differs from the other pathogens because it does not directly produce mycotoxins—it is thus considered less harmful overall [20,63].

Another difference between the silk-entering pathogens may be that *U. maydis* favours the high moisture and nutrient levels of unpollinated silks, while the other pathogens utilize more carbohydrates in pollinated silks. Varieties of maize have different levels of moisture, ash, protein, fat, and carbohydrate in the silks [77]. Overall, immature, unpollinated silks contain more fat, protein, moisture, macronutrients, and micronutrients than mature silks with fertilised, ripened grain [16]. Immature silks contain insignificantly less carbohydrates than mature silks [16].

Finally, *F. verticillioides* is an anomaly in this assembly of silk-invading pathogens, as it has endophytic properties alongside its pathogenic characteristics [36]. There are different pathogenic and endophytic strains of *F. verticillioides*, which produce differing levels of fumonisins [52]. Even in its endophytic state, *F. verticillioides* produces fumonisins [52,78]. More research concerning chemicals or maize alleles that can maintain *F. verticillioides* in an endophytic state with reduced mycotoxin production may be valuable, although complete prevention of the fungus is preferable.

## 4. Strategies to Protect Silks from Ear Rot Pathogens—Current and Future

There are general strategies currently in place to protect maize against ear rots such as improvements in crop nutrition (fertilizers), water availability, crop rotation, cropping density, tillage, fungicides, biocontrol, storage, etc., but this section will focus on specific strategies to protect the silks. Preventing contamination at silking is a prime strategy to reduce mycotoxin accumulation, as mycotoxigenic fungi introduced at silking can go on to have exponential pre- and postharvest impacts. Indeed, new silk-targeted tactics and research strategies must be developed, because the current strategies in place leave commercial maize largely susceptible. Therefore, this section introduces the strategies that already exist, including breeding for innate silk defence, agricultural inputs, and general cultural strategies. It will also discuss where these strategies fall short, and how research and development in certain areas may improve or replace these efforts.

*F. graminearum* causes more harm when infection occurs via wounds than through silks, indicating that silks have a defence mechanism against fungal infection [32]. The plant host defence system is multifaceted, which means that resistance to ear rots is based on multiple genetic traits [19]. This innate system operating in silks can help to identify specific breeding targets. Breeding programs aim to strengthen plant defences by selecting for host genetic resistance to fungal pathogens. It is important to note that silk resistance does not equate with kernel resistance (also known as seed resistance); they are two distinct types of resistance that can be selected for [48]. This review focuses on the silks, but kernel resistance is increased by characteristics such as faster drydown [73]. Separate quantitative trait loci (QTLs) have been identified for silk resistance and kernel resistance to *Fusarium* species [19,48]. It appears that there are also cob-based defences in resistant maize lines, which slow the spread of *A. flavus* down the cob [57].

The innate defence system acting in silks provides potential traits that breeders may be able to target, including metabolites and beneficial alleles, in order to protect silks against fungal pathogens. Potential breeding targets are described below.

Selection for tighter husk leaves may help to prevent insect damage to the covered portion of the silks, which subsequently reduces damage-related silk infection [79]. A plant’s first defence against infection is a physical barrier. The entranceway for fertilization is somewhat protected in many plants, such as flowers with shielding petals, and those which perform nyctinasty (close their flowers at night). However, at later stages, maize silks are exposed to the external environment, providing a gateway for incoming pathogens.

Selecting for faster silk abscission, larger abscission zones, or detachment of the silks may improve resistance. Under normal pollination circumstances, maize prevents excess pollen tubes from accessing the ovary. The abscission zone is a collapsed section in the silk channel that results soon after the first pollen tube has entered the ovule [14,80]. It occurs near the ovule, and spans 2 to 3 mm [14,80]. Maize genetics dictate the speed at which silk abscission occurs [67]. When silks are senescing and defence compounds deplete, the abscission zone may prevent pathogen entry to the kernel.

Pathogen resistance in silks can potentially be improved by developing maize lines which are less susceptible to stresses that otherwise inhibit silk growth and thus increase the time during which silks are susceptible to pathogens [81]. For example, ensuring timely pollination within a short silk emergence period has been shown to help prevent Corn Smut [60]. Breeding programs (and the natural proliferation of maize) already place importance on anthesis-silking synchrony, but it is difficult to maintain this synchrony under stressful conditions, such as when high humidity prevents pollen shed. When silk growth is inhibited, it disrupts the synchrony between anthesis and silking, and presumably silk health and defence are also affected [81]. Fortunately, the anthesis-silking interval (ASI) can be shortened using selective breeding [82]. Shortening the ASI could improve silk health when under stress, and would directly shorten the time period when silks are susceptible to pathogens. Drought is a specific stress which delays silk emergence, and thus interferes with the desired ASI [82]. Drought poses additional challenges to silks, for instance water stress decreases *F. verticillioides* growth, but increases *FUM1* (a fumonisin biosynthesis gene) transcription [83]. However, resilience of the ASI to drought stress is heritable and can be incorporated into breeding programs [82]. Furthermore, selecting for maize lines which prioritize silk extrusion relative to ear biomass (so silks still emerge under drought) has been shown to improve drought resistance [81].

Other breeding targets would include selection for an array of plant-produced defensive compounds for silk tissue, including maysin and other phenolic compounds as already noted, as well as antifungal enzymes. Maysin is a silk-produced compound which suppresses insects such as *Helicoverpa zea* (an earworm) in maize [74,84,85]. It is relevant here, because maysin helps prevent insect damage to silks, thus preventing associated (fungal) infections [79]. Silks also accumulate phenolic compounds in direct response to fungal infection [86] which could be enhanced through breeding. Indeed, the concentrations of these compounds in silks are related to plant susceptibility to pathogens [33,86]. Resistant maize genotypes react to silk infection by producing more phenolic compounds than their nonresistant counterparts, indicating the role of these compounds in innate silk defence [33,86]. Phenolic compounds oxidize to produce antifungal quinones, which are even more noxious [86]. Silks also produce various compounds with antioxidant properties, such as volatiles [87]. For example the antifungal volatile, furfural, is produced in greater quantities in *A. flavus*-resistant lines [88]. Flavonoids are nonvolatile antioxidants whose antioxidant capacity decreases as silks mature, which could relate to the increased susceptibility of aging silks to fungal colonization [16]. Baby maize and purple waxy maize have high antioxidant activity, and relatively high levels of total phenolics, flavonoids, and anthocyanins [89]. These levels vary between genotypes, which may contribute to differences in heritable resistance [89].

Additional breeding strategies could enhance antifungal proteins already present in silks including chitinases and glucanases. These enzymes may be more efficient in *A. flavus*-resistant cultivars, accounting for some of their heritable resistance [55,90]. These enzymes presumably damage the cell wall of *A. flavus*, impeding its success within silk tissue. With respect to other pathogens, maize upregulates the expression of defensive enzyme-encoding genes upon Fusarium ear rot infection [51,91]. There are gene expression studies conducted using *F. verticillioides*-infected kernels, but minimal information has been reported about gene expression in silks including antifungal proteins [51,91]. Campos-Bermudez et al. found that expression of the gene encoding glucanase is upregulated in susceptible silks in response to inoculation with *F. verticillioides* [92]. However, the authors did not detect production of chitinase in silks [92]. Lipoxygenase (LOX) genes are also associated with plant defence, and one LOX gene which is expressed in the silks, along with ZmLOX5, which may be associated with fungal resistance [51], could be selected for. Reid et al. discovered *fgsl*, a dominant (or partially-dominant) gene impacting silk resistance [93].

Additional potential breeding targets are silk-expressed genes encoding pattern recognition receptors (PRRs) to detect pathogen-associated molecular patterns (PAMPs), sometimes referred to as microbe-associated molecular patterns (MAMPs). Plant PRRs recognize *U. maydis* PAMPs, which prompt further plant defences in a system of chain reactions [51,94,95]. It would be interesting to see if this phenomenon occurs in silk tissue and can be bred.

An additional breeding strategy would be to select for prolonged silk resistance to fungal diseases. Defence compounds can only be imported into the silks or endogenously produced for a matter of days, after which the soft tissue starts to brown and decay. Maize lines bred for resistance to GER have been found also to have resistance to Corn Smut and Fusarium ear rot, so future breeding programs should focus on *F. graminearum*-resistant lines [73].

In addition to breeding, practices or inputs that increase silk health and/or maize production of defence compounds may help take advantage of the existing silk defence system to combat silk-invading fungal pathogens. For example, complimentary to the breeding approach noted earlier, farmer management practices have the potential to protect silks against drought and other stresses that otherwise increase their susceptibility to pathogens. In terms of drought, irrigation is an elementary solution, and is used in areas of the United States, China, etc. [96,97]. However, this is not practical in many areas. Weed pressure also contributes to overall crop stress [98,99], and delaying weed control was shown to directly extend the ASI in maize [99]. Therefore, appropriate weeding strategies (tillage, herbicides, and manual weeding) may improve silk health and defence [98]. High plant density can also impact ASI, and thus should be considered in the preplanting analysis of stress factors [98]. In addition, fertilizers, whether in the soil or applied as an in-season spray, may improve silk growth and health. Among the fertilizers, potassium is important for maize reproduction, as higher application rates of potassium fertilizer result in earlier tasseling and silking [100]. Presilking potassium sulphate foliar sprays have reduced early drought-induced abortions of kernels [101]. It is worth considering that applying similar micronutrient sprays during silking may improve overall silk health and hence its defence system. Calcium is another interesting nutrient in relation to silks. Calcium improves grain yield and improves resistance of maize to Corn Smut under saline conditions [102]. When pollen hydration and germination occur, calcium ion (Ca^2+^) levels in the silk trichomes (hairs) increase in a matter of seconds, and an electrical signal is propagated to the ovule [103]. This spike appears to be a form of communication within the plant, seemingly like the electrical conduction of a nerve signal. Thus, calcium is integrated in the natural functions of silks, and is another nutrient that may serve well in a late season spray. Boron deficiency results in short silks which are nonreceptive to pollen, indicating that boron is also important for silk health [104,105]. Boron has been applied as a foliar spray [104,105].

In terms of other interventions, priming agents may be developed to increase defence compound production in silks. Silica is a candidate priming agent to test on silk tissue. In maize leaves, silica can improve water use efficiency and antioxidant activity, and silica nanoparticles increase the production of defence compounds such as phenols [106,107]. Another avenue for silk protection could be polymer sprays which pose a barrier to pathogens, but not to pollen.

Fungicides are the most common chemical inputs used by growers to suppress ear rots and remain promising in terms of future research to protect silks. Significant damage and mycotoxin accumulation still occur with farmers’ current use of fungicides: it is not a complete solution. Improved, silk-specific fungicides may be useful, such as in-season sprays which are adapted to adhere to silks, or are applied as a seed coating and systemic within the silks. However, fungi can potentially develop resistance to fungicides, which increases the need for new approaches [108]. For example, *F. fujikuroi*, a fungal pathogen in rice, has developed resistance to the common fungicide phenamacril [108]. As well, fungicides employed to kill certain pathogens could create an empty niche within which competing pathogens could flourish [41]. Fungicides have a risk of producing more harm than good, for instance fungicides can induce *F. verticillioides* to produce more fumonisin B_1_ [109]. These issues with fungicides indicate a need for additional management strategies, including those that target silks.

In addition to the above strategies, biocontrol agents show promise. For example, precolonization of silks with the biocontrol fungus *Trichoderma harzianum* significantly reduces infection by *A. flavus* and aflatoxin accumulation [110]. Upcoming tactics to combat silk-infecting pathogens include silk-targeted bacterial or fungal inoculants as demonstrated by our lab [111].

Finally, examining the value of more traditional cultural practices surrounding silks could provide additional approaches to prevent fungal infestations. Traditional cultivation systems use methods which may not have been fully appreciated when maize was first scaled up to industrial production.

## 5. Concerns for the Future

Silk-entering fungal pathogens are of particular concern for the near future because of predicted disruptions associated with climate change. Pathogens which were once confined to certain latitudes and zones are now spreading to broader agriculturally relevant areas that have not faced high levels of these diseases before—bringing potential for extreme crop loss [112,113,114]. This change in pathogen distribution has been attributed to a combination of factors, including changes in rainfall, cropping patterns, and pathogen genetics [113]. These fungi have common associations with humidity and warm temperatures, which means that their geographic ranges often overlap. Silk resistance may be dependent on the climate [71], meaning that genotypes which are currently resistant may lose resiliency as climates change. These pathogens already have negative global impacts, from large commercial agrobusiness to smallholder farmers. With the oncoming climate changes, all scales of production are expected to face greater mycotoxin challenges, with disproportionate adversity coming to nations that are lacking in well-funded breeding programs and storage infrastructure.

A changing climate also means new pathogen demographics, and also a new level of damage to silk tissue, whether it is insect or weather-induced. Damage disrupts the physical barrier, allowing pathogens to enter the plant. A commonality between *F. graminearum*, *F. verticillioides*, and *A. flavus* is that damage in the field increases mycotoxin contamination. For example, weather stress can increase grain contaminated with mycotoxins produced by the latter two pathogens, specifically fumonisin and aflatoxin, respectively [42]. Since these pathogens have similar preferences and silk-infection strategies, high levels of aflatoxins can be correlated with high levels of fumonisins in maize, creating a deadly cocktail of carcinogens within a single plant [42].

There has also been an increase in popularity of no-till agriculture [115]. This creates a problem for the current control strategies in place, due to the life cycles of the invading fungal pathogens [31]. Specifically, fungal pathogens overwinter in plant debris, and no-till agriculture allows infected crop residue to persist in the environment, transmitting spores to the next growing season to attack silks [31].

## 6. Conclusions and Recommendations

Mycotoxigenic fungi are an advancing threat in our changing climate, and silks are susceptible routes of entry into the developing grain. Mycotoxins in grain cause major damage to human and livestock health and, at the very least, mycotoxins cause direct economic loss when grain cannot be consumed. Innovations are needed to defend the silk gateway into grain. Improved genetics, inputs, and cultural practices can form a better defence system to aid silks in their struggle to combat fungal pathogens. In order to develop these innovations, the research community needs to better understand the pathogens and how they interact with silk tissue. There may also be lessons to learn from nonfungal pathogens that can be detected in the silks, such as Maize Dwarf Mosaic Virus, or even from pests that attack through the silks, such as corn earworm [116].

Given the dangers of mycotoxins in today’s world, we specifically call for targeted research directed towards understanding the (1) fungal infection of silks around the pollination interval, including genes which are upregulated in the plant and the pathogen and (2) a deeper analysis of cultural practices for strategies that could be translated into conventional farming. These approaches can direct future breeding, inputs, and techniques, and challenge the pathogens which are currently gaining free passage along the maize silk road.

## Figures and Tables

**Table 1:** Maize ear rots of global importance that enter via silks and can result in mycotoxin contamination, along with the period during which maize silks are susceptible to infection.

Disease Common Name	Organism	Major Mycotoxins	Silk Susceptible Period
Gibberella Ear Rot	*Fusarium graminearum* Schw.	Deoxynivalenol, Zearalenone	After pollination, beginning of senescence, begin browning
Fusarium Ear Rot	*Fusarium verticillioides* (Sacc.) Nirenberg, *F. proliferatum* (Matsush.) Nirenberg, *F. subglutinans* (Wollenw. & Reinking)	Fumonisin B_1_	4–6 days after pollination
Aspergillus Ear Rot	*Aspergillus flavus* Link, *Aspergillus parasiticus* Speare	Aflatoxins	After pollination, beginning of senescence, yellow-brown
Corn Smut/Common Smut/Boil Smut	*Ustilago maydis* (DC) Corda, also known as *Ustilago zeae*, *Ustilago zeae mays*	No toxins ^1^	Maximum occurrence at six days after mid-silking, incidence decreasing thereafter
Diplodia Ear Rot/Stenocarpella Ear Rot	*Stenocarpella maydis* (Berk.) Sutton, previously known as *Diplodia maydis*	Diplodiatoxin	More research needed ^2^

^1^*U. maydis* is not a direct mycotoxin producer, but it can facilitate infection with mycotoxigenic ear rots. ^2^ There is minimal information on temporal periods when silks are most susceptible to Diplodia ear rot. However, in resistant cultivars silk is the most susceptible tissue.
